# Anisotropic Smoothing Improves DT-MRI-Based Muscle Fiber Tractography

**DOI:** 10.1371/journal.pone.0126953

**Published:** 2015-05-26

**Authors:** Amanda K. W. Buck, Zhaohua Ding, Christopher P. Elder, Theodore F. Towse, Bruce M. Damon

**Affiliations:** 1 Vanderbilt University Institute of Imaging Science, Vanderbilt University, Nashville, Tennessee, United States of America; 2 Department of Radiology and Radiological Sciences, Vanderbilt University, Nashville, Tennessee, United States of America; 3 Department of Biomedical Engineering, Vanderbilt University, Nashville, Tennessee, United States of America; 4 Department of Electrical Engineering and Computer Science, Vanderbilt University, Nashville, Tennessee, United States of America; 5 Department of Physical Medicine and Rehabilitation, Vanderbilt University, Nashville, Tennessee, United States of America; 6 Department of Molecular Physiology and Biophysics, Vanderbilt University, Nashville, Tennessee, United States of America; University of Minnesota, UNITED STATES

## Abstract

**Purpose:**

To assess the effect of anisotropic smoothing on fiber tracking measures, including pennation angle, fiber tract length, and fiber tract number in the medial gastrocnemius (MG) muscle in healthy subjects using diffusion-weighted magnetic resonance imaging (DW-MRI).

**Materials and Methods:**

3T DW-MRI data were used for muscle fiber tractography in the MG of healthy subjects. Anisotropic smoothing was applied at three levels (5%, 10%, 15%), and pennation angle, tract length, fiber tract number, fractional anisotropy, and principal eigenvector orientation were quantified for each smoothing level.

**Results:**

Fiber tract length increased with pre-fiber tracking smoothing, and local heterogeneities in fiber direction were reduced. However, pennation angle was not affected by smoothing.

**Conclusion:**

Modest anisotropic smoothing (10%) improved fiber-tracking results, while preserving structural features.

## Introduction

Diffusion-weighted ^1^H MRI characterizes the Brownian motion of water in tissues. This motion, the restriction thereof, and any diffusion anisotropy can be used to infer structural properties of tissues *in vivo*. In particular, a model for diffusion-weighted MRI (DW-MRI), diffusion tensor-MRI (DT-MRI) [1], can predict directional preferences in the water diffusion coefficient. DT-MRI thus can be used to characterize the structural properties of tissues with elongated cellular geometry, such as white matter tracts in the central and peripheral nervous systems and cardiac and skeletal muscle fibers [1–3]. Of particular interest to the aims of this study, DT-MRI-based muscle fiber tracking allows non-invasive estimation of three-dimensional muscle structure, such as muscle fiber pennation angle [4], fiber tract length [5], and fiber curvature [6], and allows muscle structural data to be integrated with other MR data (e.g. strain development [7]). Pennation angle (the angle formed between the fiber and the local aponeurosis surface tangent) and fiber length are related to force production [8]. Moreover, when consistent data acquisition and fiber tracking procedures are used, the fiber tract density and length may be useful for characterizing states of atrophy. Changes in these metrics related to muscle damage and repair have the potential for use in both assessing disease progression and/or response to treatment.

Challenges to acquiring high fidelity DW-MRI data include the short T_2_ of muscle, which results in low signal from the muscle, and the layer of subcutaneous fat encasing the leg, which can contribute to fat suppression difficulties. These characteristics of the tissue environment erode image quality and consequently introduce error into estimation of the diffusion tensor eigenvectors subsequently used for fiber propagation. Consequently, inferences from these data related to muscle structure and pathology may be negatively influenced. Simulations by Damon [9] demonstrated that for regions containing pure muscle, a DT-MRI signal to noise ratio (SNR) of 25 was sufficient for predicting muscle fiber orientation (within 4.5% for each component of the principal eigenvector); however, for voxels which contained a muscle fraction of 0.5, SNR requirements rose to ≥ 45. In addition, Froeling *et al*. [10] simulated the effect of low SNR on fiber tractography measures, showing that for SNR<25 fiber tract lengths were slightly underestimated (1%) and that low pennation angles were overestimated from data with low SNR.

SNR can be increased by using larger voxels and/or by increasing the number of diffusion-encoding directions or signal averages. The former approach may lead to partial volume artifacts, while the latter two approaches may be limited by total scan time and related subject motion or discomfort, particularly when studying patient and pediatric populations or when acquiring data over large volumes or multiple muscles. Although the ultimate goal should be to obtain data with high SNR, these practical limitations may limit the achievable SNR. In addition, *in vivo* images contain local data inaccuracies that impact tractography but may not be reflected in image SNR, including partial-voluming effects from intramuscular fat, which as noted by Damon [9] increases the necessary SNR for determining parameters relevant to fiber tractography, and local signal disturbances not reflected by Rician or Gaussian noise (e.g. eddy currents, B_0_ inhomogenities).

Therefore, a post-processing approach that improves fiber tractography without introducing errors through oversmoothing would be valuable. Numerous post-processing approaches to improving signal have been investigated (e.g.[11–14]); this work focuses on the use of anisotropic smoothing. Anisotropic filtering reduces noise-induced directional error in diffusion-derived parameters while preserving entity boundaries [15, 16]. Furthermore, Xu *et al*. [17] described an implementation of anisotropic filtering that considerably improves the algorithm’s efficiency. The core idea of these anisotropic filtering algorithms is to permit smoothing in regions of homogeneous tissue properties and prohibit smoothing across tissue boundaries. This is achieved by using an adaptive, anisotropic structure tensor, constructed on the basis of local image intensity profiles, to guide the direction and amount of smoothing with nonlinear diffusion equation. Although smoothing previously has been used in muscle fiber tractography [18], to our knowledge no groups have investigated the advantageous and deleterious effects of anisotropic smoothing on muscle fiber tractography.

The goal of this study was to assess the impact of an anisotropic smoothing method on fiber tractography in the medial gastrocnemius (MG) muscle in healthy subjects using DT-MRI. Increased fiber length and fiber number were considered improvements in fiber tractography, while changes in pennation angle estimation were considered symptomatic of oversmoothing.

## Materials and Methods

Healthy adult volunteers (n = 6; male = 4; mean age = 30.7 years) participated in this study, which was approved by the Vanderbilt University Institutional Review Board. Each subject provided written, informed consent before collection of data.

The subjects were positioned supine on the patient bed of a 3T Philips Achieva MR imager/spectrometer (Philips Healthcare, Best, The Netherlands). A six-channel cardiac array coil was placed around the leg. Padding was placed to prevent bulk motion of the lower extremities. After scout imaging and other setup procedures, T_1_-weighted [TR/TE = 296/6.15 ms; FOV = 192x192 mm^2^; voxel size = 0.375x0.375x6 mm^3^; NEX = 3; 24 slices] and diffusion-weighted [TR/TE = 4000/55 ms; FOV = 192x192 mm^2^; voxel size = 1.5x1.5x6 mm^3^; NEX = 5; b-value = 485 s/mm^2^; 15 diffusion encoded directions per slice; 1 non-diffusion-weighted image per slice; 24 slices] MRI data were acquired. To improve fat suppression in the DW-MRI data, these images were acquired using spectrally selective adiabatic inversion recovery (SPAIR) fat suppression (inversion time = 190 ms; 225 Hz bandwidth centered on the lipid-methylene resonance), slice-selection gradient reversal [19], and an additional 18 ms sinc-Gauss saturation pulse centered on the lipid-olefinic resonance. Each stack of 12 DW-MR slices was registered immediately after acquisition using an inline tool [20] (Philips Healthcare). For data sets acquired using these parameters, the typical SNR (determined by dividing the temporal mean of SNR for the b = 0 image by its standard deviation) was 45.

**Fig 1:**
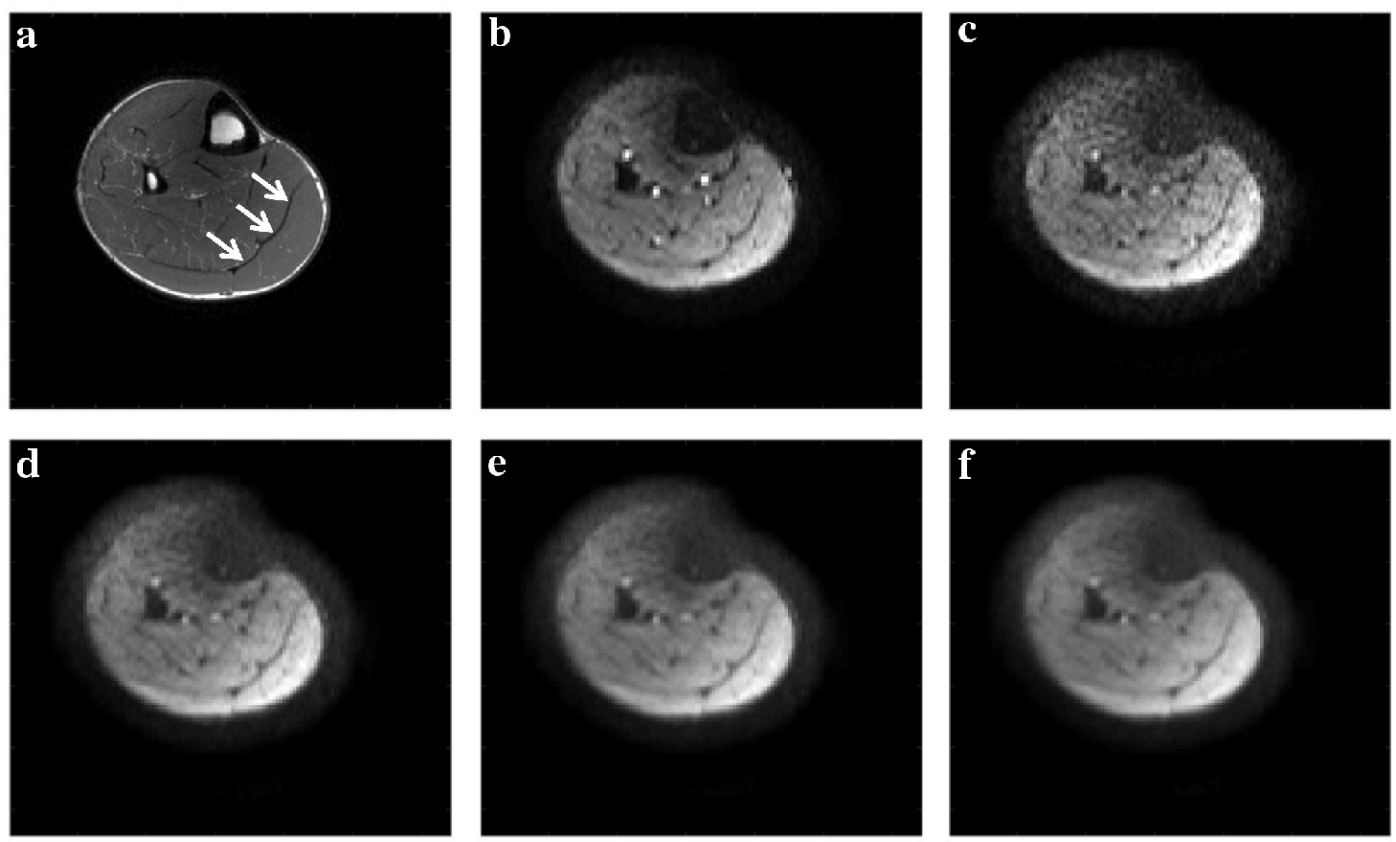
Effect of anisotropic smoothing on diffusion weighted data. Representative imaging data demonstrate (a) an anatomical image, where the white arrows indicate the location of the medial gastrocnemius aponeurosis; (b) the non-diffusion weighted image (b = 0 image); (c) the acquired diffusion weighted image encoded for the foot-head direction of diffusion. The effects of anisotropic smoothing on the foot-head encoded diffusion image is shown for (d) 5% smoothing; (e) 10% smoothing; and (f) 15% smoothing. Note that the signal loss in the anterior compartment results from the focused shimming of the posterior compartment.

MATLAB (The Mathworks, Inc., Natick MA) was used for smoothing data and for fiber tracking. Anisotropic smoothing [15], implemented with a Craig-Sneyd [17] scheme, was used in this approach [17]. According to this algorithm, in homogenous regions of a tissue, it produces near-isotropic smoothing; however, at boundaries the smoothing becomes anisotropic. This approach preserves boundary information and permits a consistent level of smoothing (anisotropic or isotropic) over the data. Please see references for additional details on the algorithm [15] and its implementation [17]. Moreover, the efficient implementation yields optimal effects with one iteration of smoothing and a step size proportional to the noise level in the original data. In this work, we anisotropically smoothed the DT-MRI data using 5, 10, and 15 times basic step sizes, which corresponded respectively to 5%, 10%, and 15% noise levels (Fig 1). The diffusion tensor and the associated eigenvectors and fractional anisotropy (FA) values were determined on a voxel-wise basis from either unsmoothed (0%) or anisotropically smoothed (5%, 10%, 15%) DT-MRI data.

**Fig 2:**
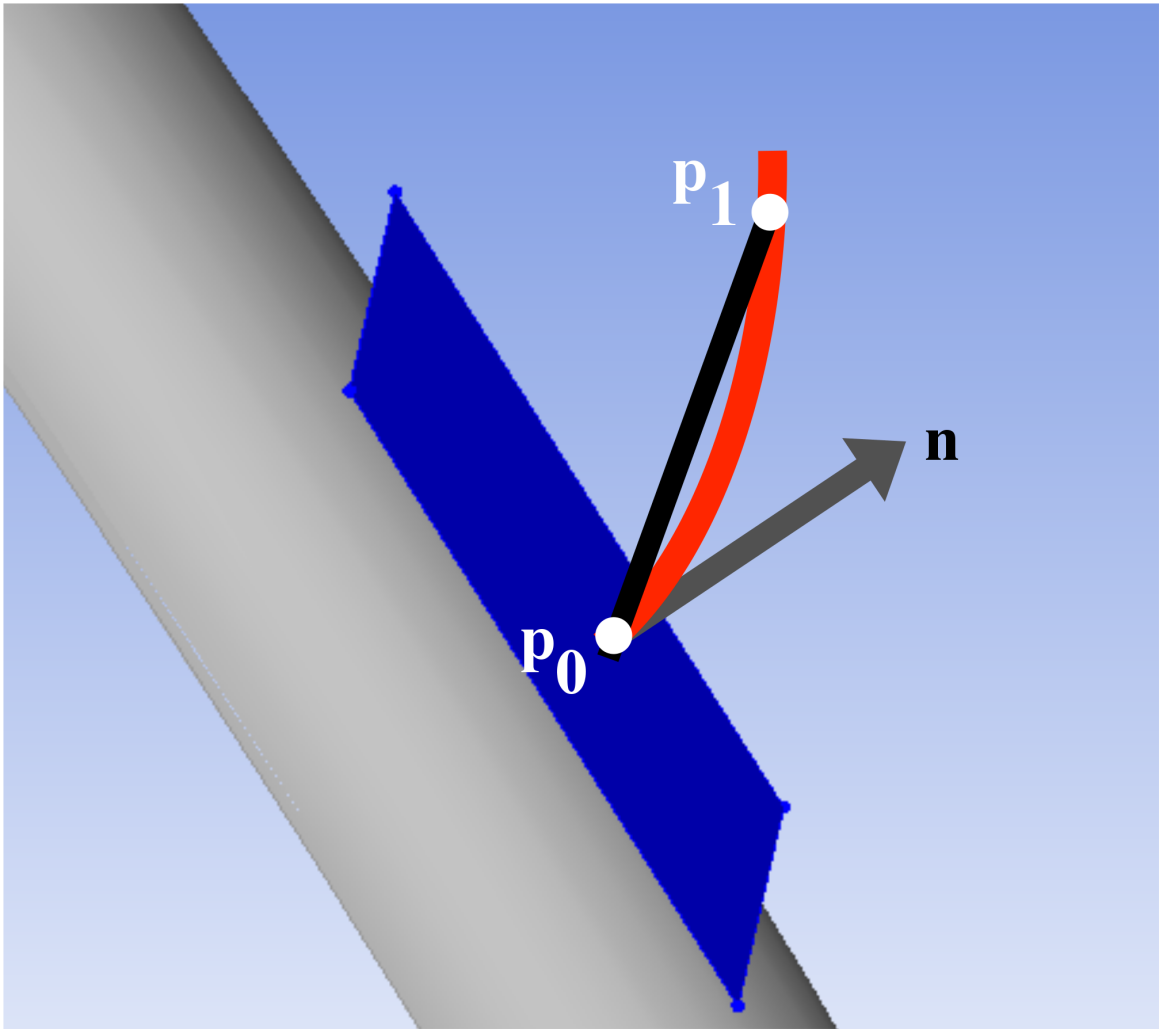
Illustration of pennation angle. The aponeurosis is represented by the grey manifold, and its tangent plane at point **p**_**0**_ is shown in blue, with its normal represented by the grey vector, **n**. For a fiber (red curve) initiated at **p**_**0**_ and tracked through point **p**_**1**_, the pennation angle is defined as the angle between the line segment defined by points **p**_**0**_ and **p**_**1**_ and the tangent plane.

For each voxel, the diffusion tensor, **D,** was estimated using weighted least squares regression [21] of the signal intensities on the 15-direction diffusion encoding matrix. **D** was diagonalized and the eigenvalues were magnitude-sorted. The voxel-wise FA was calculated from the eigenvalues of **D**, as described previously [22]. To determine the MG boundary, the b = 0 DT-MR images were segmented using an intensity-based approach (www.itksnap.org) [23]. Each medial gastrocnemius aponeurosis was traced over the 20 most distal slices of the segmented muscle and was rendered as a three-dimensional rectangular mesh (described by a 50×100 array). To accomplish the fiber tracking using a streamline approach [2, 24], tracts were initiated from the aponeurosis grid points and were propagated based on principal eigenvector orientation if 1) the tract remained within the muscle boundary; 2) the current voxel FA value was between 0.05 and 0.35; and 3) the angle formed between two adjacent eigenvectors was <20°. Fiber number was determined as the number of fibers that were tracked at least one step from the seed points on the aponeurosis. Following the work of Lansdown et al. [25], the pennation angle is defined between the local aponeurosis tangent plane at the fiber seed point and the line extending between the seed point (p_0_) and the first tracked point (p_1_) of the same tract (Fig 2).

For each statistical comparison of interest, a two-sided Wilcoxon rank sum test with a Bonferroni correction of the critical p-values was used to determine statistical significance (p<0.01) between smoothing levels.

## Results

**Fig 3:**
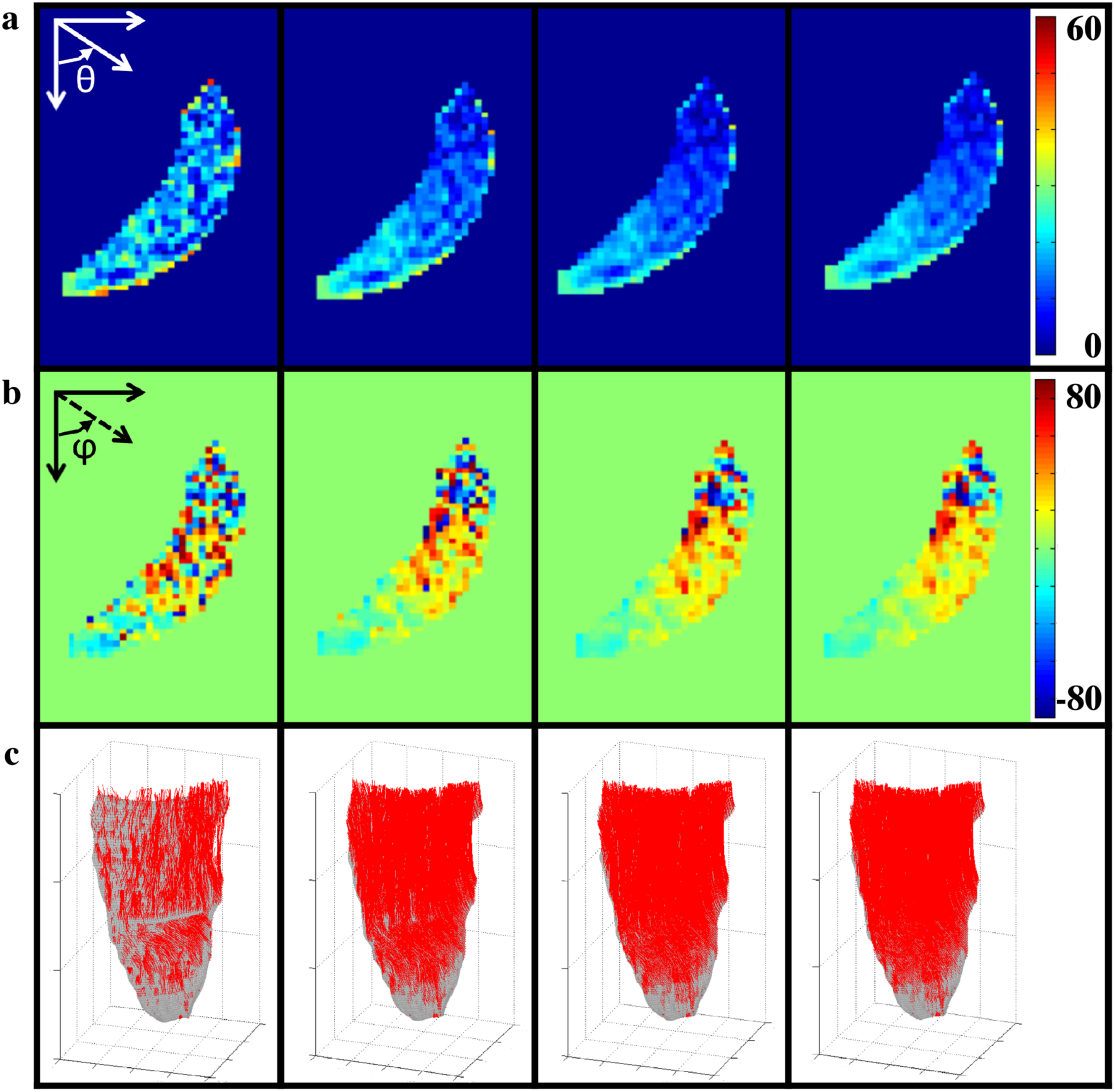
Effects of anisoptropic smoothing on principal eigenvector heterogeneity. Modest smoothing moderates heterogeneity of the principal eigenvector as shown by (a) polar angle (θ) for 0%, 5%, 10%, 15% smoothing (left to right); and (b) azimuthal angle (φ) for 0%, 5%, 10%, 15% smoothing (left to right) for a representative axial slice in the leg. (c) Resulting fiber tracks (red) for the entire MG muscle of the same subject displayed on the aponeurosis mesh (grey) for 0%, 5%, 10%, 15% smoothing levels (left to right).

Anisotropic smoothing of the diffusion data impacted the number and length of tracked fibers and the number of fibers that extended the length of the masked volume. In addition, local heterogeneities in the estimated fiber orientation decreased, as shown in representative maps describing the polar (θ) and azimuthal (φ) angles of the principal eigenvector (Fig 3a and 3b). Further, mean FA values within the muscle masks were significantly lower for the 10% and 15% smoothed data than for unsmoothed data (Table 1).

**Table 1:** Median and (range) for FA, pennation angle, number of fibers, and fiber length.

	0%	5%	10%	15%
**FA**	0.240 (0.236–0.261)	0.216 (0.081–0.243)	0.211* (0.190–0.236)	0.206^ (0.190–0.231)
**Pennation Angle (°)**	19.54 (16.68–27.59)	18.82 (16.58–26.34)	18.79 (16.49–25.38)	18.86 (16.39–25.38)
**Number of fibers**	1179 (357–2424)	2722* (1487–3675)	2838^#^ (2126–3644)	2874^#^ (2126–3643)
**Fiber length (mm)**	16.82 (14.34–28.19)	33.66^ (28.96–41.98)	35.21^ (30.74–41.48)	35.36^ (30.50–41.19)

* indicates a statistical difference (p = 0.009) from unsmoothed (0%) data for the group;

^^^ indicates a statistical difference (p = 0.0022) from unsmoothed (0%) data for the group;

^#^ indicates a statistical difference (p = 0.0043) from unsmoothed (0%) data for the group.

Implementing the smoothing algorithm improved the fiber tract sets, although the mean pennation angle was unaffected (Table 1). Smoothing significantly increased the number of fibers tracked and the mean fiber tract length; although there were no significant differences between the 5%, 10%, and 15% smoothing levels (Table 1; Fig 3c). In addition, smoothed data resulted in fewer tracts prematurely stopping due to the angle curvature criteria, consequently increasing the number of tracts that stopped propagating at the muscle boundaries. However, the number of fibers stopping due to going beyond the prescribed FA range did not change significantly with smoothing (Table 2).

**Table 2:** Median number of fibers and (range) that terminated from each stop criteria.

	0%	5%	10%	15%
**Outside FA threshold**	**570**	**356**	**442**	**460**
	**(237–1264)**	**(188–572)**	**(231–769)**	**(219–788)**
**Exceeded curvature threshold**	**3386**	996*	298*	112*
	**(2918–4011)**	**(109–2782)**	**(8–1582)**	**(0–1582)**
**Tract exited mask**	**1067**	3750*	4343*	4454*
	**(357–1369)**	**(1752–4497)**	**(2882–4572)**	**(2882–4731)**

* indicates a statistical difference (p = 0.0022) from unsmoothed (0%) data for the group.

## Discussion

In this paper we have demonstrated that anisotropic smoothing of DT-MR data for muscle fiber tractography improved fiber tracking results, reduced noise-related bias and preserved physiologic characteristics related to muscle function.

Several characteristics of the data indicate improved fiber tracking results. First, the number of fiber tracts extending to the muscle boundaries increased; consequently, so too did the mean length of the tracked fibers. This is not an indication of longer fascicle length, rather it is a measure of the extent of fiber tract propagation through the data set. Second, the orientation of muscle fibers smoothly varies across the MG (i.e. there are not large changes in direction of neighboring fibers). Thus, the decrease of local heterogeneity in first eigenvector orientation, as presented in Fig 3, represents a more physiological architecture (i.e. fiber direction is smoothly varying across the cross-section of the muscle). The smoothing-related decrease of principal eigenvector local heterogeneity reduced the number of tracts that stopped prematurely due to high curvature in adjacent pairs of points in a tract. This decrease in fiber direction heterogeneity is similar to the results of Xu *et al*. (2010) who demonstrated recovery of the principal diffusion direction with anisotropic smoothing of noise corrupted simulated data; further the approach was shown to decrease the effect of noise on estimates of diffusion parameters for human *in vivo* brain data [17]. In addition, Ding *et al*. (2005), demonstrated that this anisotropic smoothing approach outperformed Gaussian smoothing in maintaining the principal direction of diffusion, particularly as the number of smoothing iterations increases [15]. The smoothly varying, simple architecture of muscle fiber alignment in the medial gastrocnemius is approached at low levels of smoothing, in contrast to the more complex and rapidly varying orientation of brain white matter structures. It is noteworthy that these reductions in the heterogeneity of the first eigenvector occurred despite an SNR level (45) that was sufficient, as predicted by the simulation studies of Damon [9, 10] and Froeling *et al*. [10].

Since oversmoothing can introduce error into parameter estimations, we investigated the effect of varying smoothing levels on the pennation angle and other fiber tract characteristics. Our results showed that pennation angle is unaffected by anisotropic smoothing. Although Froeling *et al*. [10] predicted that pennation angle asymptotically approaches its true value with increased SNR, it is likely that the data in the current study has sufficient SNR that the pennation angle is unaffected by smoothing. Since the approach preserves features at the structure boundaries [16, 17] and the pennation angles are by definition found at these boundaries, this demonstrates the utility of this approach in muscle tractography. Further, the absence of statistically significant differences in fiber tract length or in number of fibers successfully tracked with an increase in smoothing level suggests that less aggressive smoothing is sufficient to improve the tracking. In consideration of 1) computational processing time, and 2) the findings of Xu *et al*. (2010), who reported that filtering human *in vivo* data at less aggressive smoothing levels proved more effective at decreasing error [17], we recommend a modest level of smoothing (10%) for MRI-based muscle tractography.

A decrease in mean FA value over the muscle ROI was observed with smoothing; however, since the mean values remain well within the range required for fiber tract propagation, the number of tracts halted due to exceeding the FA threshold was not significantly impacted by smoothing. The FA shows a trend (though no statistical significance) of further decrease with additional smoothing, echoing the findings of Xu *et al*. (2010) [17] in human subject DTI data from the brain. This trend also may be related to the finding of Basser and Pajevic (2000) that bias in eigenvalue magnitude is inversely related to SNR [26]; thus, the smoothing may be reducing the bias inherent with noise. Further, Oouchi *et al*. (2007) demonstrated that FA is underestimated in areas of crossing white matter fibers in the brain when larger, anisotropic voxels are used [27]. However, in the current study, physiological structure of the muscle fibers in the region studied is largely homogeneous with smoothly varying fiber directions, such that anisotropy of voxels is unlikely to introduce appreciable error. Since potential applications include the use of muscle fiber tractography in patients with disease, it is important to note that anisotropically smoothed data performed better than Gaussian (isotropic) smoothed data at detecting pathology based on FA thresholding [28], and Ding *et al*. demonstrated that an anisotropic approach is superior to that of Gaussian smoothing when assessing FA [15]. In addition, FA errors are reduced with anisotropic smoothing relative to Gaussian filtering, and they are relatively stable even when using sub-optimal parameters [16].

It should be emphasized that performance of the efficient anisotropic smoothing depends on the step size, which can be determined from a reasonable estimation of noise level in the data to be smoothed. Thus, if the structures of interest have varying levels of noise, a trade-off in the step size has to be made to yield overall optimal smoothing effects.

In conclusion, a modest level (10%) of anisotropic smoothing will preserve physiological features of fiber tracts while improving muscle fiber tractography.
